# Correlation Between Screen Time, Sleep Duration, and Stroop Effect Among First-Year Osteopathic Medical Students: A Cross-Sectional Study

**DOI:** 10.7759/cureus.109106

**Published:** 2026-05-18

**Authors:** Raju Panta, Pedro Del Corral, Summer Hales, Piercarla Fernandez, Emily Ahearn, Mark Parsamian

**Affiliations:** 1 Physiology and Pathology, Burrell College of Osteopathic Medicine, Melbourne, USA; 2 Physiology and Pathology, Burrell College of Osteopathic Medicine, Las Cruces, USA; 3 Osteopathic Medicine, Burrell College of Osteopathic Medicine, Las Cruces, USA; 4 Osteopathic Medicine, Burrell College of Osteopathic Medicine, Melbourne, USA

**Keywords:** cognitive function tests, executive function, medical students, reaction time, screen time, selective attention, sleep duration, stroop effect, stroop interference, stroop test

## Abstract

Background: The Stroop test is a well‑validated measure of selective attention and inhibitory control. Although increased electronic device use has been consistently associated with shorter sleep duration and delayed bedtime, whether everyday variability in screen time and sleep relates to Stroop performance in medical trainees remains uncertain, and existing findings on executive function are mixed.

Objective: This study aimed to examine associations between average daily screen time, self‑reported sleep duration (past week and past month), and Stroop performance measured using the EncephalApp (congruent reaction time, incongruent reaction time, and Stroop interference) in first-year osteopathic medical students.

Methods: In this cross‑sectional study conducted between March and July 2025, 69 first‑year osteopathic medical students were recruited, of whom 49 (10 male, 39 female) provided complete datasets. Participants completed the EncephalApp Stroop test on their smartphone and an anonymous survey reporting demographic characteristics, average daily screen time over the past week, and sleep duration over the past week and past month. Simple linear regression analyses were used to examine associations between Stroop interference and screen time, Stroop interference and sleep duration, and between screen time and sleep duration.

Results: Participants (n = 49; 10 male, 39 female) had a mean age of 25.61 ± 3.58 years. The mean congruent reaction time (OffTime) was 54.06 ± 10.06 seconds, the incongruent reaction time (OnTime) was 60.94 ± 13.59 seconds, and the mean Stroop interference score was 6.87 ± 7.38 seconds. The average daily screen time over the past week was 6.10 ± 3.12 hours, while the average sleep duration was 6.93 ± 1.26 hours over the past week and 7.04 ± 1.16 hours over the past month. Stroop interference was not significantly associated with screen time (R² = 0.0023; p = 0.746) or sleep duration (past week: R² = 0.0287; p = 0.250; past month: R² = 0.0303; p = 0.237). However, screen time was inversely associated with sleep duration over the past week (R² = 0.14; β = −0.152 hours of sleep per additional hour of screen time; p = 0.0088).

Conclusions: Among first-year osteopathic medical students, typical variation in screen time and sleep duration was not associated with Stroop‑indexed selective attention. Higher screen time was, however, moderately associated with shorter sleep duration. These findings are consistent with prior evidence linking electronic media use to reduced sleep and suggest that modest between‑person differences in sleep and screen exposure may not translate into measurable differences in Stroop performance in this population.

## Introduction

The Stroop effect, characterized by slower and/or less accurate responses to incongruent color-word stimuli compared with congruent or neutral stimuli, is a well‑established measure of selective attention and inhibitory control [1]. For example, when the word “RED” is printed in blue ink, individuals typically require more time to name the ink color than when the word and color are congruent, illustrating the additional processing demands imposed by cognitive conflict [1]. Cognitive neuroscience research indicates that Stroop interference arises across multiple processing stages, with substantial involvement of lateral and medial prefrontal networks. Ongoing theoretical debate centers on the relative contributions of top‑down executive control versus stimulus‑driven attentional processes in mediating this interference [1,2].

Concurrently, the widespread use of light‑emitting electronic devices has raised concerns regarding their impact on sleep duration and circadian regulation. In this context, screen use refers to engagement with digital devices, such as smartphones, tablets, computers, and televisions, while screen time denotes the total duration of daily exposure to these devices. Large systematic reviews and consensus statements consistently report that greater screen use is associated with shorter sleep duration, delayed bedtimes, and increased sleep disturbances, with effect sizes varying by study design, age group, and measurement approach [3-5]. These associations are directionally consistent across populations: higher screen exposure predicts reduced total sleep, later sleep onset, and poorer sleep continuity. Proposed mechanisms include suppression of nocturnal melatonin secretion by blue‑enriched light and increased cognitive or emotional arousal that delays sleep onset [3,4]. “Recent evidence further suggests that these effects are moderated by factors such as timing, intensity, screen content, and individual susceptibility [6,7].

With respect to executive performance, experimental sleep restriction reliably impairs vigilance and several executive domains, with shorter sleep duration generally associated with slower reaction times, reduced accuracy, and diminished cognitive control [8]. However, findings specific to Stroop performance remain mixed across acute sleep‑deprivation and short‑sleep paradigms. Studies in college‑aged populations have reported that reduced sleep duration and poorer sleep quality are associated with longer Stroop reaction times and higher error rates, indicating greater cognitive interference under sleep‑restricted conditions [9]. More recent neuroimaging and functional near‑infrared spectroscopy (fNIRS) studies in young adults further suggest that severe short sleep selectively impairs accuracy on incongruent trials and is associated with altered prefrontal activation patterns, reflecting increased neural effort during conflict processing [10,11].

Medical students represent a population at heightened risk for sleep disturbance due to heavy cognitive demands, frequent evening study, and substantial reliance on electronic devices. The existing literature indicates that sleep deprivation negatively affects academic performance in medical students, with poor sleep quality consistently associated with lower academic outcomes and elevated stress levels [3,12]. Although some studies have not demonstrated a direct relationship between sleep duration and academic performance, they nonetheless identify strong links between poor sleep quality and increased stress [12].

Despite this evidence, it remains unclear whether typical between‑person variation in screen time and sleep duration among first‑year medical trainees is associated with selective attention as measured by Stroop interference, defined as the additional time required to respond to incongruent color-word stimuli relative to congruent stimuli, reflecting the cognitive cost of resolving conflict between automatic word reading and goal‑directed color naming. Stroop interference is widely used as an index of inhibitory control and prefrontal executive functioning, and prior work suggests that it is sensitive to factors such as fatigue, sleep loss, and cognitive load. Accordingly, this study examined whether (1) screen time and (2) sleep duration over the past week and past month predicted Stroop interference, and separately, whether (3) screen time was inversely associated with sleep duration. We hypothesized that greater screen time and shorter sleep duration would be associated with increased Stroop interference, and that screen time would be inversely related to sleep duration.

## Materials and methods

Study design and setting

A cross‑sectional observational study was conducted between March and July 2025 at Burrell College of Osteopathic Medicine, across both the New Mexico and Florida campuses. The study was designed to examine associations between lifestyle factors (screen time and sleep duration) and selective attention as measured by Stroop interference in first-year medical students.

The study protocol was reviewed and approved by the Institutional Review Board of Burrell College of Osteopathic Medicine prior to data collection (IRB #0151_2024). All study procedures complied with ethical standards for human subject research. Participation was voluntary, and all data was collected anonymously.

Participants and recruitment

Participants were recruited using a non‑probability convenience sampling approach, also known as voluntary response sampling, in which all first‑year osteopathic medical students were invited to participate through institutional announcements and electronic communication. Interested students accessed an electronic informed consent form and, upon consent, completed study procedures remotely using their personal smartphone devices and an online survey platform.

A total of 69 students consented to participate. Of these, 49 participants provided complete Stroop task data along with complete survey responses and were included in the final analysis. No incentives or compensation were offered for participation.

Eligibility criteria

Participants were eligible for inclusion if they were enrolled as first‑year osteopathic medical students at Burrell College of Osteopathic Medicine (BCOM) during the 2025 academic year, were able to download and complete the EncephalApp Stroop Test on a personal smartphone (Android or iOS), and complete an anonymous online survey reporting demographic information, average daily screen time, and sleep duration for the preceding week and/or month. Eligibility further required complete paired data for Stroop performance, including congruent (OffTime) and incongruent (OnTime) reaction times, as well as corresponding screen‑time and sleep‑duration measures.

Participants were excluded if they withdrew consent, failed to complete study procedures, or provided incomplete data, including missing Stroop task results, screen‑time reports, or sleep‑duration measures. Additional exclusions included failure to complete the Stroop test due to technical issues or partial participation, incomplete survey responses that precluded calculation of key variables, and self‑reported uncorrected visual or color‑vision impairment that interfered with task performance. A total of twenty participants were excluded due to incomplete datasets, most commonly resulting from missing Stroop performance data or incomplete reporting of sleep or screen‑time variables.

Sample size calculation

Sample size estimation was performed in advance using Cochran’s formula for finite populations [13], based on an estimated population of approximately 300 first‑year osteopathic medical students at BCOM. Using a 95% confidence level, a conservative proportion estimate of 50%, and a 5% margin of error, the minimum required sample size was calculated and then adjusted for the finite population. An additional inflation factor was applied to account for anticipated non‑response, yielding a target sample size of 185 participants. This target reflected the requirements for a population‑level prevalence estimate, not the analytic needs of the regression models. Although the final analyzable sample (n = 49) was substantially smaller due to voluntary participation and incomplete datasets, it remained sufficient for the planned simple linear regression analyses because these models involved only one predictor at a time, the observed effect sizes were small, and the achieved sample size exceeded commonly cited minimums (n ≈ 30-50) for stable estimation in single‑predictor regression. Accordingly, while the reduced sample limits the precision of population estimates, it was adequate for detecting moderate associations and addressing the study’s analytic objectives.

Measures

Stroop Task and Cognitive Performance

Selective attention and cognitive interference were assessed using the EncephalApp Stroop Test (Android/iOS), a validated mobile‑based version of the classic Stroop Color-Word paradigm that demonstrates strong test-retest reliability and criterion validity for measuring psychomotor speed and cognitive flexibility [14]. Participants completed the assessment on their personal smartphones in a self‑directed but standardized format.

Before testing, participants configured the application using uniform settings, including enabling instructional guidance, activating subject identification for in‑app storage only, and setting the number of practice and recorded trials. Each participant completed two practice trials followed by five recorded trials for both congruent (Stroop Off) and incongruent (Stroop On) conditions.

In the congruent condition (OffTime), participants selected the ink color of neutral symbols (#) displayed in red, blue, or green. In the incongruent condition (OnTime), participants identified the ink color of color words (“RED,” “BLUE,” or “GREEN”) presented in conflicting ink colors (e.g., the word “RED” printed in blue ink). The application automatically recorded the total completion time in seconds for each condition.

The extracted Stroop performance metrics included reaction time during the congruent condition (OffTime), reaction time during the incongruent condition (OnTime), the total Stroop completion time calculated as the sum of OffTime and OnTime, and a Stroop interference score computed as the difference between OnTime and OffTime. All reaction‑time values were recorded in seconds (s). In the EncephalApp scoring framework, faster completion times indicate better psychomotor speed and attentional control, whereas larger interference scores reflect greater difficulty resolving cognitive conflict during incongruent trials. Normative data from validation studies demonstrate OffTime and OnTime values typically in the range of tens of seconds in healthy adults, with interference scores remaining positively skewed, where higher values correspond to increased cognitive interference [14].

Participants manually entered their recorded Stroop results into the anonymous Qualtrics survey. Subject identifiers remained stored only within the app on the participant’s device and were not shared with investigators, and investigators did not have access to any raw Stroop performance data within the EncephalApp itself beyond the values voluntarily entered by participants.

Screen Time and Sleep Assessment

Screen time and sleep duration were assessed via an anonymous Qualtrics survey. Participants reported demographic information (age and gender), average daily screen time over the preceding week (hours per day), average nightly sleep duration over the preceding week (hours per night), and average nightly sleep duration over the preceding month.

Participants were encouraged to reference device-generated screen‑time and sleep‑tracking data (e.g., smartphone digital wellbeing or health applications) when available. For consistency, all reaction times are reported in seconds (s), and all screen‑time and sleep‑duration measures are reported in hours (h).

The survey also included items used to confirm inclusion and exclusion criteria. No personally identifiable information was collected. The complete questionnaire, including all original and translated survey items used to assess screen time, sleep duration, demographic variables, and eligibility criteria, is presented in the Appendix. This instrument was developed by the authors specifically for this study.

Data Management and Confidentiality

All submitted data were deidentified at the time of collection. Survey responses and Stroop performance variables were stored in a password‑protected Microsoft Excel file accessible only to study investigators. Data will be securely retained for three years following completion of the study and then permanently deleted in accordance with institutional policy.

Statistical Analysis

Data were imported into Microsoft Excel (Microsoft Corp., USA) for data cleaning and statistical analysis. Prior to conducting inferential tests, the distribution of continuous variables was examined using visual inspection of histograms and descriptive measures to assess approximate normality. Descriptive statistics were calculated to summarize participant demographics, Stroop performance measures, average daily screen time, and sleep duration. To evaluate the study hypotheses, all statistical tests, including the series of simple linear regression analyses, were performed in Microsoft Excel, examining Stroop interference (OnTime-OffTime) as a function of average daily screen time, average sleep duration over the past week, and average sleep duration over the past month, as well as average sleep duration over the past week as a function of screen time. Regression outputs included slope coefficients, coefficients of determination (R²), and corresponding p-values. No additional statistical software was used. All statistical tests were two‑tailed, with statistical significance defined as p < 0.05.

## Results

Participant characteristics and task performance

A total of 49 first‑year osteopathic medical students, 10 males and 39 females, provided complete datasets and were included in the final analysis. Additional demographic variables collected included age, vision problems not corrected by glasses, and color‑vision problems. Because no participants reported color‑vision deficiencies and only a minority reported uncorrected vision problems, these variables were not included in Table 1. Descriptive statistics for age, Stroop performance, screen‑time exposure, and sleep duration are presented in Table 1.

**Table 1:** Descriptive statistics (N = 49) Screen time refers to the average daily screen use reported for the preceding week. Sleep duration represents the self‑reported average nightly sleep for the preceding week and month. Stroop interference was calculated as the incongruent reaction time (OnTime) minus the congruent reaction time (OffTime). In this table, s denotes seconds and h denotes hours.

Parameter	Mean ± SD
Age (years)	25.61 ± 3.58
Congruent Stroop (OffTime, s)	54.06 ± 10.06
Incongruent Stroop (OnTime, s)	60.94 ± 13.59
Stroop Interference (On–Off, s)	6.87 ± 7.38
Average screen time, last week (h/day)	6.10 ± 3.12
Average sleep duration, last week (h/night)	6.93 ± 1.26
Average sleep duration, last month (h/night)	7.04 ± 1.16

The participants had a mean age of 25.61 ± 3.58 years, as indicated in the descriptive output (mean age = 25.61). Stroop task performance followed the expected pattern, with longer reaction times during incongruent trials relative to congruent trials. The mean congruent reaction time (OffTime) was 54.06 ± 10.06 seconds, and the mean incongruent reaction time (OnTime) was 60.94 ± 13.59 seconds, yielding a mean Stroop interference score of 6.87 ± 7.38 seconds. These values align with the dataset summary (OnTime minus OffTime duration: mean = 6.87; SD = 7.38) and reflect modest but variable cognitive slowing during conflict resolution across participants.

**Figure 1:**
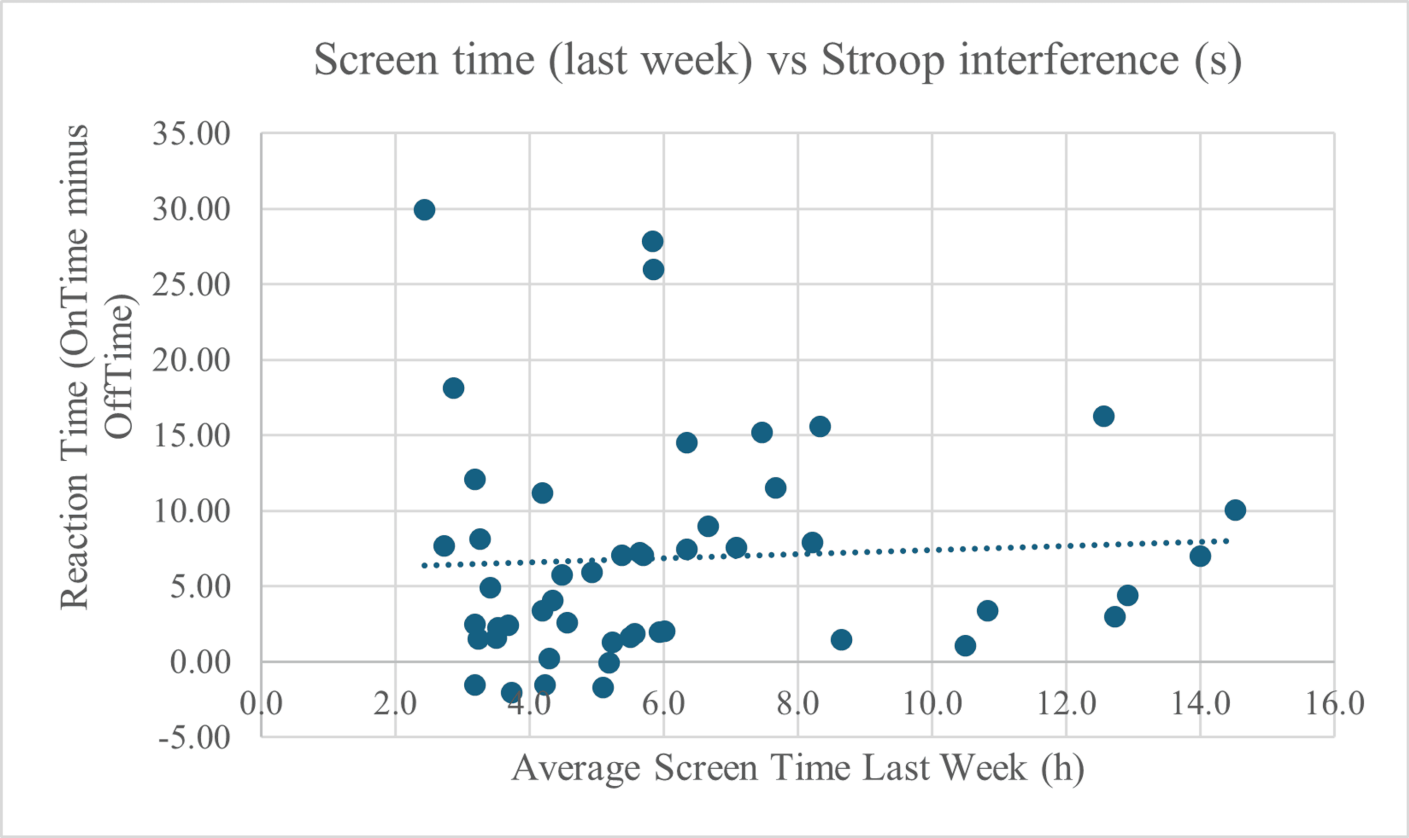
Screen time (last week) vs. Stroop interference (s) (N = 49) Scatter plot displaying the average daily screen time (hours per day) during the preceding week and Stroop interference (OnTime−OffTime, seconds). Simple linear regression line shown.

**Figure 2:**
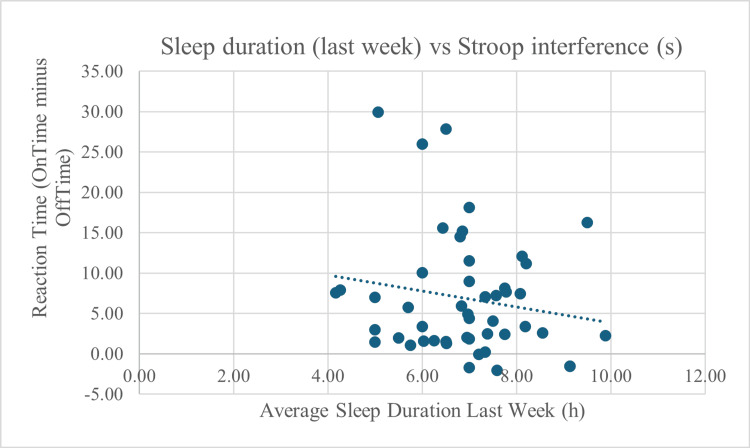
Sleep duration (last week) vs. Stroop interference (s) (N = 49) Figure 2. Scatter plot displaying average nightly sleep duration (hours per night) during the preceding week and Stroop interference (OnTime − OffTime, seconds). Simple linear regression line shown.

**Figure 3:**
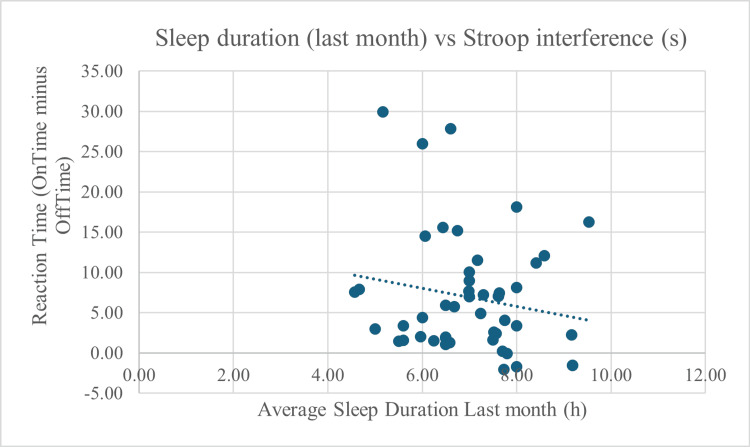
Sleep duration (last month) vs. Stroop interference (s) (N = 49) Scatter plot displaying the average nightly sleep duration (hours per night) during the preceding month and Stroop interference (OnTime − OffTime, seconds). Simple linear regression line shown.

**Figure 4:**
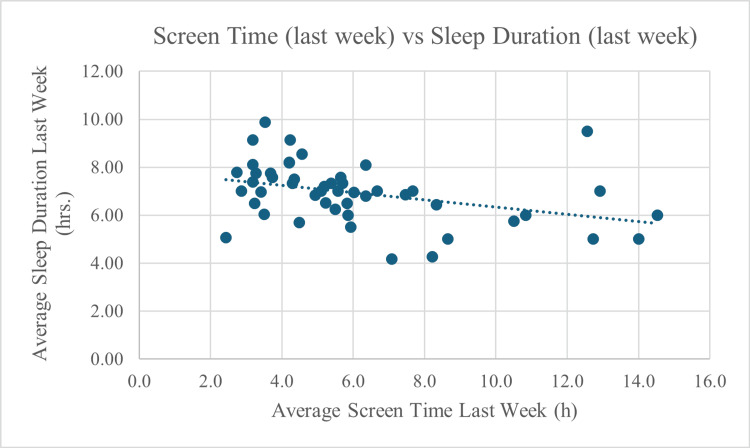
Screen time (last week) vs. sleep duration (last week) (N = 49) Scatter plot displaying average daily screen time (hours per day) and average nightly sleep duration (hours per night) during the preceding week. Simple linear regression line shown.

With respect to lifestyle measures, the participants reported a mean daily screen time of 6.10 ± 3.12 hours over the preceding week (Table 1). The mean nightly sleep duration was 6.93 ± 1.26 hours for the past week and 7.04 ± 1.16 hours for the past month (Table 1). Figures 1-4 illustrate the distribution and variability of these behavioral measures across the participants.

Bivariate correlations among the screen time, sleep duration, and Stroop interference

Preliminary Pearson correlation coefficients were calculated prior to regression analyses (Table 2). Screen time demonstrated no meaningful correlation with Stroop interference (r = 0.048), consistent with the negligible variance explained in the regression model. Sleep duration showed small, nonsignificant negative correlations with Stroop interference for both the past week (r = −0.169) and the past month (r = −0.174).

**Table 2:** Correlation and linear regression results Correlation and linear regression results examining associations among screen time, sleep duration, and Stroop interference. Pearson correlation coefficients (r) were derived from the corresponding simple linear regression models by taking the square root of R² and assigning the sign of the regression slope (β). The 95% confidence intervals (CI) reflect the precision of each estimated regression coefficient. Negative values indicate inverse associations between variables. All analyses were two‑tailed, with statistical significance defined as p < 0.05.

Predictor → outcome	R²	Correlation (r)	Slope (β)	95% CI for β	p-value	Interpretation
Screen time (last week, h) → Stroop interference (s)	0.0023	+0.048	+0.113 s/h	−0.581 to +0.807	0.7456	No meaningful effect
Sleep (last week, h) → Stroop interference (s)	0.0287	−0.169	−0.975 s/h	−2.63 to +0.68	0.250	Trend negative, not significant
Sleep (last month, h) → Stroop interference (s)	0.0303	−0.174	−1.089 s/h	−2.92 to +0.74	0.237	Trend negative, not significant
Screen time (last week, h) → Sleep (last week, h)	0.1400	−0.374	−0.152 h/h	−0.264 to −0.040	0.0088	Moderate negative association

By contrast, screen time and sleep duration (past week) exhibited a moderate inverse correlation (r = −0.374), indicating that higher screen exposure was associated with shorter nightly sleep. This pattern mirrors the significant regression finding reported below (Table 2).

Associations between Stroop interference, screen time, and sleep duration

Screen Time and Stroop Interference

Simple linear regression revealed no significant association between average daily screen time and Stroop interference (R² = 0.0023, p = 0.7456). As shown in Figure 1, the scatterplot demonstrates a wide dispersion of interference scores across all levels of screen exposure, with no discernible linear trend. The slope of the regression line was near zero, indicating that greater screen use did not correspond to increased cognitive interference. These results are summarized in Table 2.

Sleep Duration (Last Week) and Stroop Interference

Average nightly sleep duration over the preceding week was also not significantly associated with Stroop interference (R² = 0.0287, p = 0.250). As illustrated in Figure 2, although the regression line shows a slight negative slope, suggesting numerically lower interference among participants with longer sleep duration, the relationship was weak and not statistically significant. The modest downward trend did not account for meaningful variance in interference scores, as reflected in the low R² value. Corresponding regression statistics are presented in Table 2.

Sleep Duration (Last Month) and Stroop Interference

Similarly, sleep duration over the past month did not significantly predict Stroop interference (R² = 0.0303, p = 0.237). Figure 3 shows a pattern comparable to the weekly sleep model, with a shallow negative slope and substantial variability across participants. The regression model explained only 3% of the variance in interference scores, indicating that typical month‑to‑month differences in sleep duration were not associated with measurable changes in selective attention or inhibitory control. Full regression results are provided in Table 2.

Screen Time (Last Week) and Sleep Duration (Last Week)

In contrast to the null findings for Stroop performance, a statistically significant inverse association was observed between average daily screen time and average nightly sleep duration over the preceding week (R² = 0.14, p = 0.0088). As shown in Figure 4, participants with higher screen exposure tended to report shorter sleep duration. The regression slope (β = −0.152 hours per additional hour of screen time) indicates that each additional hour of daily screen use corresponded to an estimated nine‑minute reduction in nightly sleep. This model accounted for 14% of the variance in weekly sleep duration, representing a moderate effect size relative to the other models examined. These findings are detailed in Table 2.

## Discussion

In this cohort of first-year osteopathic medical students, no significant associations were identified between Stroop interference scores and either self‑reported sleep duration or average daily screen time, indicating that the degree of cognitive interference measured by the EncephalApp Stroop task remained relatively stable across variations in these lifestyle factors. These findings suggest that, within the typical ranges of sleep and screen use observed in this population, selective attention and inhibitory control may be preserved, and modest fluctuations in these behaviors may not exert measurable effects on Stroop‑based cognitive performance.

By contrast, the analysis revealed a moderate and statistically significant inverse relationship between screen time and sleep duration over the preceding week, with higher daily screen exposure corresponding to shorter nightly sleep. This trend aligns with contemporary evidence from systematic reviews and consensus statements demonstrating that increased electronic media use is consistently associated with reduced sleep duration, delayed bedtimes, and greater sleep disruption across diverse populations. Such patterns are often attributed to a combination of evening cognitive stimulation, behavioral displacement of sleep, and light‑mediated suppression of melatonin [4]. The present findings reinforce these established associations and highlight the relevance of screen‑related behaviors as a modifiable factor influencing sleep health in medical student populations [3,4].

Screen time and sleep duration

In the present study, the inverse association between screen exposure and nightly sleep, equivalent to an estimated nine-minute reduction in sleep per additional hour of screen time and accounting for 14% of the variance in sleep duration, is consistent with findings from large‑scale meta‑analyses and epidemiologic investigations. Multiple reviews have demonstrated that increased screen use is associated with shorter total sleep duration, delayed bedtimes, and difficulty initiating sleep across broad age groups [3,4]. For example, a 2025 systematic review and meta‑analysis involving over 548,000 participants found that each added hour of screen time was linked to three to five minutes less total sleep, along with delayed bedtimes averaging 13.2 minutes per hour of exposure and increased odds of insomnia symptoms [4]. Similarly, updated analyses of electronic media use have shown that digital device engagement is significantly associated with reduced sleep quality and a higher prevalence of sleep disturbances across multiple populations [7].

Mechanistic explanations for these effects include both light‑mediated circadian disruption and behavioral or emotional arousal. Short‑wavelength (blue‑enriched) light emitted by smartphones and other LED‑based displays has been repeatedly shown to suppress nocturnal melatonin secretion, delay circadian phase, and disrupt subsequent sleep physiology [3-5]. Studies confirm that evening smartphone use, even for as little as 90 minutes, significantly attenuates melatonin levels in young adults and reduces deep sleep (N3) in the early night, reinforcing recommendations to avoid device exposure before bedtime [15-18]. The existing evidence suggests that while higher screen time, especially in the evening, is associated with both reduced sleep and delayed sleep timing at the population level, within‑person daily variation may exert its most meaningful influence on bedtime timing rather than total sleep duration. This distinction highlights the importance of factors such as timing, content, and context of device use, and it underscores why targeted behavioral interventions (e.g., limiting screen use in the hour before bed) continue to be emphasized in clinical sleep‑health recommendations [18-20].

Moreover, a 2024 literature review on blue‑light exposure in adolescent and college‑aged populations highlights the extent to which modern light‑emitting technologies contribute to circadian dysregulation and diminished sleep quality. These physiological effects parallel the longstanding sleep‑hygiene guidance issued by major sleep‑health organizations, including the American Academy of Sleep Medicine (AASM) and the National Sleep Foundation (NSF), both of which caution against evening screen use due to its potential to disrupt melatonin release and natural sleep timing [3,19,21].

A newer within‑person analysis provides a more nuanced interpretation of the relationship between screen use and sleep. Rather than uniformly reducing total sleep time, daily fluctuations in screen exposure appear to be more strongly associated with sleep timing, specifically delayed sleep onset [11,12]. A 2026 systematic review and meta‑analysis of daily screen use in youth found that higher‑than‑usual screen time on a given day reliably predicted a later bedtime, while showing minimal impact on total sleep duration, sleep efficiency, or subjective sleep quality. These findings have been independently replicated across additional within‑person studies, which similarly report that day‑to‑day screen use primarily shifts sleep timing rather than reducing overall sleep quantity [17].

Sleep duration and Stroop performance

Contrary to our initial hypothesis, sleep duration did not significantly predict Stroop interference in this cohort. This finding suggests that modest, naturally occurring between‑person differences in habitual sleep, in the order of approximately one hour, may be insufficient to produce measurable variation in selective attention or inhibitory control as assessed by the Stroop task. Our results stand in contrast to experimental research showing that substantial or acute sleep restriction reliably impairs executive functioning, including inhibitory control, decision‑making, and vigilance. For example, controlled sleep‑deprivation paradigms demonstrate significant declines in cognitive abilities and executive function, accompanied by alterations in neural mechanisms such as metacognitive monitoring and inhibitory processing under fatigue [10]. These neurophysiological findings are further supported by the other studies documenting that disrupted sleep can impair prefrontal cortex‑mediated executive functions and modulate event‑related potentials associated with interference tasks like the Stroop [22-24].

However, such impairments are typically observed under conditions of total sleep deprivation or severe sleep restriction, which differ substantially from the habitual sleep ranges reported by our participants. For example, meta‑analytic and experimental evidence shows marked executive‑function deficits, including impaired Stroop performance, primarily under substantial sleep loss rather than mild nightly variation [16,19,25].

Indeed, prior studies in college‑aged and young adult populations provide mixed evidence regarding associations between sleep metrics and Stroop outcomes. Some investigations have reported correlations between actigraphically measured sleep and Stroop performance, such as increased incongruent errors or longer reaction times among individuals with shorter or less efficient sleep [11], while others have found opposite or null associations [10].

For instance, one study demonstrated that sleep efficiency was significantly associated with Stroop incongruent errors and simple reaction time, whereas sleep duration itself was not consistently related to interference performance [22]. Another investigation found that young adults with short sleep exhibited altered prefrontal activation and reduced accuracy on incongruent Stroop trials, suggesting that the cognitive impact of insufficient sleep may depend more on the severity and chronicity of restriction than on minor night‑to‑night variability [15].

Taken together, these heterogeneous findings indicate that while experimental or severe sleep restriction reliably produces robust impairments in executive functioning, including deficits in inhibitory control, decision‑making, and cognitive efficiency, as demonstrated in controlled deprivation and neurophysiological studies [26,27], between‑person variability in typical sleep duration-such as the modest differences observed in our sample, may not reliably translate into measurable differences in Stroop interference among healthy young adults. Evidence from collegiate samples further supports this interpretation: although some studies have identified associations between sleep metrics and Stroop performance, findings remain inconsistent, with results varying by direction, magnitude, and task sensitivity. For example, one actigraphy‑based study reported mixed associations, with sleep efficiency predicting Stroop incongruent errors while sleep duration did not consistently relate to interference scores [28,29]. Likewise, neuroimaging‑supported work has shown that only severe short sleep (<6 hours) is linked to reduced accuracy and altered hemodynamic responses during incongruent Stroop trials, effects not observed in those with typical sleep ranges [11].

Such discrepancies highlight how methodological variability, including differences in sample sizes, sleep measurement approaches (subjective vs. objective), and Stroop paradigms, contributes to divergent outcomes across literature. Systematic reviews and meta-analyses emphasize that the use of multiple experimental protocols, diverse cognitive batteries, and heterogeneous outcome definitions adds further complexity to interpreting sleep‑related executive deficits [4,7,17].

Consequently, our findings align with a growing body of evidence suggesting that mild variability in habitual sleep does not significantly impair performance on brief, well‑practiced executive tasks, such as the EncephalApp Stroop, particularly within healthy, high‑functioning young adult populations [11,28,29].

Stroop mechanisms

Reconciling the null behavioral associations in our study with well‑established neurocognitive models of Stroop performance, it is plausible that first‑year medical students engage compensatory control mechanisms that allow them to maintain stable behavioral performance despite routine variability in sleep or screen exposure. Neuroimaging evidence demonstrates that lateral prefrontal cortex (PFC) regions bias attention toward task‑relevant stimulus features and suppress competing information, thereby supporting successful performance on interference tasks such as the Stroop. Medial prefrontal regions, by contrast, contribute more heavily to response‑related and late‑stage control processes. Crucially, these regions interact in a cascade‑like manner, with early control processes influencing the degree of later control needed to resolve conflict, providing a neural explanation for why behavioral Stroop interference may remain stable even when upstream cognitive resources fluctuate [1,2].

Furthermore, contemporary cognitive models emphasize that interference in the Stroop task reflects multiple processing loci, spanning stimulus encoding through response selection. Under this framework, behavior may appear unaffected even when neural effort increases, a characteristic pattern in highly trained or high‑capacity individuals, such as medical students. Empirical work using functional MRI demonstrates that distributed activation across frontal regions, including lateral PFC, anterior cingulate cortex (ACC), and associated networks, predicts individual resilience to interference and supports compensatory adjustments that maintain performance under varying cognitive demands [30].

Thus, the preserved Stroop performance observed in our cohort is consistent with cascade‑of‑control models and stimulus‑driven selective attention accounts, which predict that individuals with sufficient cognitive reserve may recruit additional prefrontal resources to compensate for minor perturbations in sleep or attentional state, preventing measurable deficits at the behavioral level [17,18].

Strengths and practical implications

A methodological strength of this study is that the observed effect sizes for the associations between screen time, sleep duration, and Stroop interference were extremely small (R² < 0.03). Although our final sample (n = 49) was smaller than the conservative target derived from Cochran’s formula, standard power analyses for simple linear regression indicate that samples of approximately 55-70 participants are typically required to detect medium‑sized effects with adequate power. Because our sample size fell slightly below this threshold, the study would have been underpowered only for detecting effects of medium magnitude or larger. However, the negligible effect sizes observed here suggest that the nonsignificant findings are unlikely to be attributable solely to limited power. Practically, this implies that typical between‑person variation in sleep and screen exposure among first‑year medical students may not meaningfully influence Stroop‑indexed selective attention, even in larger samples.

This study has several methodological strengths. First, it leverages objective timing from a standardized mobile Stroop platform, ensuring consistency in the administration and scoring of cognitive interference measures across participants. Second, the sample consists of a focused cohort of first‑year medical trainees, reducing heterogeneity related to age, educational level, and occupational demands, which are the factors that commonly confound cognitive and sleep research in broader populations. Third, the use of convergent sleep windows (past week and past month) strengthens the reliability of self‑reported sleep duration and improves interpretability regarding recent versus habitual sleep patterns.

From a practical perspective, the findings meaningfully support institutional wellness messaging aimed at improving sleep health among medical trainees. Although habitual sleep variability did not translate into detectable group‑level differences in Stroop interference, the demonstrated relationship between higher screen exposure and shorter sleep duration underscores the value of promoting healthier digital habits. This aligns with AASM recommendations, which emphasize limiting screen use, particularly in the 30-60 minutes before bedtime, to reduce circadian disruption and protect sleep quality [21]. Furthermore, consensus statements from the National Sleep Foundation, synthesizing results from more than 2,200 scientific studies, affirm that screen use, especially pre‑bedtime content, impairs sleep health and that behavioral strategies such as moderating evening device use can mitigate these effects [3].

Thus, even in the absence of measurable cognitive performance differences, the present results reinforce evidence‑based wellness guidance encouraging students to reduce evening screen exposure as a means of protecting sleep, an established priority in sleep‑health promotion.

Limitations

Several limitations warrant consideration. First, the cross‑sectional design prevents any determination of causality. Although associations between screen exposure, sleep, and cognitive performance were examined, the temporal ordering of these variables cannot be established. In addition, residual confounding is possible, as potentially relevant factors-such as chronotype, the timing and content of device use, caffeine consumption, academic workload, or stress‑related behaviors-were not directly measured or included in the analytic models. These unmeasured variables may have influenced both sleep patterns and cognitive performance.

Second, both screen time and sleep duration were primarily self‑reported, which introduces recall bias and limits measurement precision. Objective tools such as device‑based usage logs, ecological digital‑tracking metrics, or actigraphy‑derived sleep estimates would provide more accurate quantification of exposure and sleep-wake behavior. Incorporating objective timing of device use, particularly in the hours preceding bedtime, would further refine the characterization of associations.

Third, the sample size was relatively small (n = 49) and fell below the estimated sample size needed to detect medium‑sized effects, which may limit statistical power. Although the observed effect sizes were extremely small, the reduced sample size still constrains the precision of estimates and increases the likelihood of type II error. The sample was also predominantly female (39 of 49 participants) and drawn from a single institution and academic year, which further limits generalizability. Sleep behavior, screen‑exposure habits, and cognitive responses may differ across sex, training environments, or stages of medical education. Broader, multi‑institutional samples would strengthen external validity.

Fourth, Stroop performance values were manually entered by participants after completing the EncephalApp task. Although the app provides clear numerical outputs, manual transcription introduces the possibility of entry errors or minor inaccuracies. Direct extraction of performance metrics from the application or automated data capture would reduce this source of measurement error.

Finally, the Stroop interference metric, while widely used as an index of selective attention and inhibitory control, may be less sensitive than other executive‑function measures for detecting subtle between‑person cognitive differences within a healthy, high‑performing cohort. Tasks such as the n‑back working‑memory paradigm, task‑switching measures, or response‑inhibition tasks (e.g., Go/No‑Go or Stop‑Signal) often show greater sensitivity to small variations in executive control. Small effect sizes may therefore require larger samples, more demanding executive tasks, or tasks that probe sustained or complex control processes to identify meaningful associations.

Future directions

Future investigations should incorporate objective wearable technologies, including actigraphy or advanced sensor‑based devices, to capture sleep duration, timing, and regularity with greater precision. Objective sleep measurement is especially valuable given that actigraphy‑derived sleep parameters have proven sensitive to intervention‑related changes in prior randomized studies examining the impact of screen exposure on sleep efficiency and night awakenings [31]. Similarly, the adoption of passive sensing systems capable of quantifying screen exposure timing, luminance, and content would meaningfully reduce recall bias and enable more ecologically valid assessments of digital behavior in medical trainees.

To more rigorously probe within‑person dynamics, future studies should employ time‑of‑day-controlled cognitive testing, ensuring that Stroop performance is measured at consistent circadian phases and that potential fluctuations in alertness or sleep pressure are minimized. In parallel, experimental manipulations, such as randomized control trials that adjust the timing and content of evening screen use, could help clarify causal pathways linking digital behaviors to sleep and executive function. Evidence from randomized clinical trials manipulating pre‑bedtime screen exposure in younger populations has already demonstrated the feasibility of such interventions and their capacity to alter objectively measured sleep parameters [31].

Finally, digital hygiene interventions, including sleep‑focused behavioral programs delivered via web‑ or app‑based platforms, represent a promising direction for medical trainees. Systematic reviews of digital sleep interventions in young adults highlight their potential for improving sleep outcomes using scalable, technology‑supported approaches [32]. Embedding such interventions into medical training programs may offer a practical pathway to mitigate sleep disruption and support cognitive performance in high‑demand academic environments.

## Conclusions

This study examined whether typical variation in screen exposure and sleep duration among first‑year osteopathic medical students relates to selective attention as measured by Stroop interference. Within this cohort, selective attention appeared relatively stable across the range of sleep and screen‑use behaviors reported, suggesting that modest day‑to‑day differences in these lifestyle factors may not meaningfully influence Stroop‑indexed inhibitory control in this population.

At the same time, the observed pattern of screen use and sleep duration underscores the relevance of technology‑related behaviors for sleep health in medical trainees, a group already vulnerable to sleep disruption due to academic demands. These findings highlight the importance of continued attention to sleep‑supportive habits in medical education and point to the need for future research using larger samples, objective behavioral measures, and longitudinal designs to clarify how lifestyle factors interact with cognitive performance over time.

## Appendices

**Table 3:** Original and translated survey questionnaire This questionnaire is the authors’ original creation. No external copyrighted instrument was used; therefore, no permissions were required.

Original text	Translated text
Consent	Consent
The researcher has explained that this research is to study the correlation between screen time, sleep duration, and Stroop effect among first-year osteopathic medical students.	This study examines the relationship between screen time, sleep duration, and Stroop test performance among first‑year osteopathic medical students.
I know the details about the procedure of the Stroop Test, collection of data on average screen time and sleep duration for last week and last month from my mobile device for the purpose of the research, and the time commitment.	I understand the Stroop Test procedure, the collection of screen‑time and sleep‑duration data from my mobile device, and the associated time commitment.
I have understood the issues of confidentiality.	I understand how my confidentiality will be protected.
I know that my participation is voluntary, and I will try to answer all questions and comply with the required tests to the best of my ability.	I understand that participation is voluntary and that I may withdraw at any time.
I voluntarily agree to participate in this study.	I agree to participate.
I do not agree to participate in this study.	I do not agree to participate.
Age	Age
What is your age?	Please indicate your age in years.
Gender	Gender
How do you identify?	Please select your gender identity.
Man	Man
Woman	Woman
Prefer to self-describe	Prefer to self-describe
Exclusion/Inclusion – Questionnaires	Eligibility Questions
Do you have any vision problems not corrected by glasses?	Do you have uncorrected vision problems?
Yes / No	Yes / No
Do you have any color vision problems?	Do you have color vision difficulties?
Yes / No	Yes / No
Stroop effect	Stroop effect
The Stroop test results as duration in seconds	Record Stroop test durations in seconds.
Congruent Stroop (OffTime duration):	Congruent condition (OffTime) duration in seconds:
Incongruent Stroop (OnTime duration):	Incongruent condition (OnTime) duration in seconds:
OffTime plus OnTime duration:	Total Stroop duration (OffTime + OnTime):
OnTime minus OffTime duration:	Stroop interference score (OnTime − OffTime):
Screen time	Screen time
Average screen time of last week:	Average daily screen time during the past week:
Hours / minutes	Hours / minutes
Sleep duration	Sleep duration
Average sleep duration of last week:	Average nightly sleep duration during the past week:
Hours / minutes	Hours / minutes
Average sleep duration of last month:	Average nightly sleep duration during the past month:
Hours / minutes	Hours / minutes
